# Research Progress of pH-Responsive Indicator Films for Real-Time Food Quality Monitoring

**DOI:** 10.3390/foods15193566

**Published:** 2026-10-08

**Authors:** Zhijie Li, Xincheng Duan, Rongxin Wu, Jianchao Wang, Zhiyong Yang, Danfei Liu

**Affiliations:** School of Packaging Engineering, Hunan University of Technology, Zhuzhou 412007, China; 23404100107@stu.hut.edu.cn (Z.L.); 2408040101035@stu.hut.edu.cn (X.D.); 2408170301006@stu.hut.edu.cn (R.W.); 2408170301025@stu.hut.edu.cn (J.W.); 2408170301031@stu.hut.edu.cn (Z.Y.)

**Keywords:** pH-responsive indicator film, natural dyes, meat monitoring, freshness, metal–organic frameworks

## Abstract

Facing global food waste and safety challenges, real-time nondestructive quality monitoring via intelligent packaging has gained substantial interest. This narrative review critically examines the progress of pH-responsive freshness indicator films, with a particular emphasis on their application to meat and meat products, and emphasizes the transition from empirical exploration toward rational design. Methodologically, we synthesize recent advances in natural pigment-based indicators, focusing on their fabrication strategies (micro/nanoencapsulation, metal–organic framework immobilization, pigment blending, and matrix engineering) and performance evaluation models integrating multivariate statistical tools (principal component analysis, cluster analysis, and discriminant analysis). The results reveal that replacing synthetic dyes with anthocyanins and betalains significantly mitigates the inherent toxicity risks, while advanced immobilization and blending strategies substantially overcome poor stability and high dye migration, achieving synergistic improvements in sensitivity, anti-interference, and mechanical durability. Furthermore, establishing a comprehensive evaluation framework linking total volatile basic nitrogen accumulation to color-change thresholds is crucial for accurate interpretation. We conclude that the field is shifting from single-function validation toward multi-signal responsiveness, digital integration, and systematic implementation. It should be noted, however, that the colorimetric response of these indicators reflects quality attributes such as TVB-N accumulation rather than the presence of pathogens or toxins; therefore, these indicators cannot independently establish food safety. Future breakthroughs require addressing environmental robustness, long-term reliability, standardized evaluation protocols, and scalable cost-effective production to facilitate the transition from laboratory research to practical supply chain deployment.

## 1. Introduction

In an era of growing global concern over food safety and sustainable development, effectively reducing food waste and ensuring the quality and safety of perishable foods constitute a core challenge in food science [1]. Globally, approximately 1.3 billion tonnes of food are estimated to be wasted or lost each year, with meat and meat products representing a substantial proportion of this total (e.g., around 23% by some estimates) [1]. Fresh meat, rich in moisture and protein, is highly susceptible to microbial proliferation, posing a potential threat to public health. This contradiction highlights the urgent need for real-time, non-destructive quality monitoring of food, especially meat products [2].

However, traditional detection methods are characterized by significant drawbacks, including delayed response, destructiveness, and high cost. These conventional approaches for assessing meat freshness, which are based on chemical and microbiological properties (e.g., measurement of pH, total volatile basic nitrogen (TVB-N), thiobarbituric acid reactive substances (TBARS), and total viable count (TVC)), rely heavily on specialized instrumentation and destructive sampling. They suffer from inherent limitations such as cumbersome operation, long detection cycles, and inaccurate evaluation, and they require trained personnel, making them difficult to embed within the modern, fast-moving food supply chain [3,4,5]. Such limitations—time lag, destructiveness, and high expense—prevent producers, retailers, and consumers from obtaining accurate, real-time information on meat freshness. This gap, in turn, directly drives a paradigm shift in the functionality of food packaging: from providing passive physical protection toward an intelligent stage capable of autonomous sensing, information communication, and even active regulation [6].

Against this backdrop, intelligent packaging technology has emerged. Building upon the physical protection afforded by conventional packaging, it further integrates advanced technologies such as biotechnology, electronics, sensors, and the Internet of Things, thereby endowing packaging with the capacity for real-time sensing and characterization of food quality. As an integrated system that combines detection, sensing, recording, and tracking functions, intelligent packaging can monitor in real time the internal and external environmental changes and quality dynamics experienced by fresh agricultural products during supply chain circulation, thus enabling early warning of potential risks and providing a more transparent and continuous information dimension for the entire chain [7]. Intelligent packaging not only protects the product but also delivers real-time response signals to changes in packaging conditions and food quality, primarily through technologies such as sensors, indicators, and radio frequency identification (RFID) [8]. Among the various types of intelligent packaging, freshness indicator packaging—which can directly respond to specific biochemical signals generated during food spoilage (e.g., volatile amines, sulfides, or pH changes) and display them in the form of intuitive color changes—exhibits considerable application potential [9]. In particular, for meat spoilage, a process typically characterized by protein degradation and a rise in pH, pH-responsive indicator films offer a monitoring approach that is direct in principle and relatively low in cost. Meat is rich in fat and protein and is susceptible to oxidation and microbial contamination during storage; a substantial quantity of meat is discarded and wasted each year due to spoilage in the supply chain, resulting in enormous economic losses [10]. In this context, intelligent food packaging capable of real-time detection of meat freshness and extension of shelf life can help mitigate meat waste in the supply chain, empower consumers to accurately access quality information, and hold significant importance for regulating the food market order and safeguarding consumer rights.

However, the current development and application of this technology remain largely based on synthetic chemical dyes (e.g., methyl red, bromocresol purple, and methylene blue). Methylene blue, for instance, has been employed not only as a redox indicator for spoilage metabolite detection but also in detecting defects in metal oxide-coated food packaging materials. Although such dyes offer the advantage of high sensitivity, their potential biotoxicity and environmental impact are increasingly at odds with the modern food industry’s pursuit of safety, greenness, and sustainability, thereby posing an inherent bottleneck to technology adoption [9]. In the field of pH-sensing indicator development, numerous studies have focused on immobilizing pH-sensitive dyes using natural biodegradable polymers, including bacterial cellulose nanofibers, chitosan, and starch–cellulose composites [11,12,13]. Among these, chitosan, as a non-toxic and renewable biopolymer, possesses the ability to form highly transparent films. Consequently, exploring the construction of novel indicator systems based on safe and non-toxic natural active pigments (e.g., anthocyanins and betalains), and combining them with advanced techniques such as nano-encapsulation and metal–organic frameworks to overcome inherent drawbacks such as poor stability and susceptibility to migration, has emerged as a frontier research direction for breaking through the current predicament [14].

This transformation concerns not only the safety upgrade of detection technologies but also entails the integration of knowledge across the entire chain, from a deep understanding of indicator principles and precise modeling of the spoilage process to the systematic innovative design of responsive material systems. Accordingly, this review aims to critically evaluate the research progress on intelligent packaging materials for real-time food quality monitoring, with a particular focus on pH-responsive freshness indicator films, and to explore the core pathways driving their transition from empirical exploration to rational design. We first elucidate the spoilage mechanisms of meat as a representative food matrix and the fundamental working principles of corresponding indicators. Subsequently, we delve into how integrated microbiological, chemical, and environmental factors can be harnessed to establish a comprehensive freshness change model, thereby providing a quantified theoretical benchmark for indicator film design. Building on this, we focus on the core material innovation pathway from synthetic dyes to natural dyes, analyzing how materials compounding and structural design can be leveraged to enhance their performance. Specifically, this review is structured around three core themes: (1) the chemical mechanisms of meat spoilage and the working basis of pH response; (2) the construction methodologies for comprehensive freshness evaluation models and the logic of threshold calibration; and (3) functional enhancement strategies for natural dyes and the optimization of indicator film material systems.

While several recent reviews have addressed the development of natural pigment-based indicator films [9,15,16], and a growing body of work from European and North American groups has also contributed to the understanding of indicator chemistry and packaging applications [6,8,17,18], the present review distinguishes itself by offering three distinctive contributions. First, we propose a unified “spoilage-to-signal” framework that explicitly connects meat spoilage biochemistry (microbial proliferation → TVB-N accumulation → pH rise) to the design parameters of indicator films, thereby providing a rational foundation for threshold calibration that is largely absent in previous overviews. The classification and functional roles of different intelligent packaging indicators are summarized in Table 1. Second, we provide a systematic evaluation of the three major functional enhancement strategies for natural dyes—micro/nano-encapsulation, MOF immobilization, and pigment blending—with a comparative assessment of their respective strengths, limitations, and practical applicability (summarized in Table 2 and Table 3), moving beyond the descriptive summaries that characterize much of the existing literature. Third, we introduce a multivariate statistical framework (PCA–CA–DA) as a standardized protocol for calibrating color-response thresholds against a comprehensive spoilage state, an aspect that has received little attention in previous reviews. In contrast to the predominantly material-centric focus of earlier works, our review emphasizes the critical but often overlooked interface between spoilage chemistry, statistical validation, and material design, with the goal of facilitating the translation of laboratory research into practical supply-chain applications.

Finally, based on the above analysis, we systematically prospect the challenges and future development trends in this field, with the goal of providing a clear academic vision and actionable research insights for the development of safer, more efficient, and practical intelligent food packaging systems.

## 2. Principles of Food Freshness Indication

### 2.1. Classification and Functional Orientation of Intelligent Packaging Systems

Intelligent food packaging is a multidimensional, interdisciplinary concept; its core lies in endowing conventional packaging with information monitoring and interaction functions through the integration of chemical, physical, or biological sensing units. Based on differences in their core functions and response mechanisms, intelligent packaging can be primarily categorized into time–temperature indicators, freshness indicators, and gas indicators, which collectively constitute the technical spectrum of food quality monitoring.

Time–temperature indicators (TTIs) represent a classic type of intelligent packaging component, operating on the principles of chemical reaction kinetics. A temperature-sensitive chemical or enzymatic reaction system is typically encapsulated within the indicator; as time and temperature accumulate, the reaction products build up irreversibly and induce progressive changes in color or morphology [26,27]. Consumers can observe these visual changes with the naked eye to judge whether the food has deteriorated, thereby avoiding health risks associated with the consumption of spoiled food. The core advantage of TTIs lies in their ability to intuitively reflect whether the product has experienced inappropriate temperature fluctuations during storage and transportation—that is, they monitor the “thermal history” of the cold chain. Their fundamental limitation, however, is that they indicate the “input” of environmental stress rather than the “output state” of food quality deterioration. The ultimate spoilage of food results from the coupled effects of microbial activity, endogenous enzyme action, and the characteristics of the food matrix, with temperature being only one contributing factor [17]. Consequently, TTIs are more suitable for simple systems in which the spoilage rate exhibits a highly linear correlation with temperature.

Freshness indicators (FIs), in contrast to the indirect monitoring of TTIs, are designed to directly “sniff out” the characteristic metabolites generated during food spoilage, thereby reflecting the intrinsic quality status of the food in real time [15]. Their working principle is based on the chemical recognition of specific spoilage metabolites (e.g., amines, sulfides, ammonia, or organic acids), which are released by microbial metabolism or the food’s own biochemical reactions, with their accumulated concentrations exhibiting a high correlation with the degree of quality deterioration. When the target compound reaches a critical concentration within the packaging, it triggers a visual color change in the indicator system, thereby transforming the invisible spoilage process into an intuitive visual signal [18].

Through the color change in freshness indicators induced by specific food volatiles, FIs enable rapid assessment of food freshness [28]. Their unique advantage lies in providing qualitative or semi-quantitative information on food quality deterioration caused by microbial growth or physiological and biochemical changes, without compromising the integrity of the food package, thereby allowing consumers to intuitively and scientifically evaluate the true state of the food [29]. This characteristic of directly targeting the internal quality state of food positions freshness indicators as the most promising technological pathway for achieving real-time, in situ quality monitoring, and this topic constitutes the core focus of this review.

Gas indicators (MGIs), by comparison, are functionally oriented toward monitoring the physicochemical parameters of the internal packaging microenvironment (e.g., O_2_/CO_2_), rather than the food spoilage products themselves. During actual storage and transportation, maintaining the quality stability of food within the packaging system is a complex proposition involving multiple variables, including the respiration rate of fresh produce, changes in gas concentrations, the gas barrier properties of the packaging material, and gas production by microbial metabolism. Gas indicators are designed to address these microenvironmental regulation challenges by generating a color change in response to specific chemical or enzymatic reactions, thereby providing real-time information on the concentration of oxygen or carbon dioxide within the package [30]. In modified atmosphere packaging (MAP) applications, the scientific regulation of gas ratios and precise temperature control are key factors in ensuring the effectiveness of freshness preservation: if the carbon dioxide concentration is excessively high, it dissolves in the water of fruit and vegetable tissues to form carbonic acid, which alters the acidity and flavor of the food and may even cause physiological disorders. Thus, gas indicators function by monitoring changes in the concentration of key gases (such as O_2_ or CO_2_) in the packaging headspace to provide early warning of packaging seal failure or abnormal respiratory metabolism of the food. Their function has a clear environmental orientation and serves as important technical support for maintaining the stability of a specific packaging microenvironment; however, unlike FIs, they do not directly reflect the degree of food spoilage.

In summary, within the technical spectrum of intelligent packaging, the three categories of indicators assume distinct functional roles: TTIs trace the environmental history, FIs sense food quality, and MGIs monitor the packaging microenvironment. To more clearly illustrate their synergies and distinctions, Table 1 provides a systematic comparison in terms of principle, monitoring target, advantages and limitations, and application scenarios. Among these, freshness indicators, by virtue of their unique functional positioning of directly correlating with the core aspect of food quality, hold irreplaceable value for constructing a quality transparency system oriented toward the end consumer.

### 2.2. Chemical Nature of Meat Spoilage and Key Quality Indicators

Designing effective freshness indicators necessitates a deep understanding of the chemical nature of food spoilage—the very target being monitored. Taking fresh meat as an example, its deterioration is a complex process dominated by microorganisms and driven by the synergistic action of multiple biochemical reactions. Post-mortem muscle sequentially passes through the stages of rigor mortis, resolution of rigor, and aging, accompanied by the dynamic interplay of lactic acid accumulation from anaerobic glycolysis and the generation of alkaline products from protein decomposition. Consequently, the pH of meat exhibits a characteristic pattern of first decreasing and then increasing: it typically declines to its minimum value (approx. pH 5.5) at 12–24 h post-mortem, and subsequently rises slowly due to the continuous accumulation of alkaline metabolites, potentially exceeding pH 6.5 at the spoilage stage. After the innate immune barriers are lost, muscle tissue becomes a natural medium for microbial growth [16]. Under suitable conditions, the initially contaminating microorganisms (mainly Pseudomonas spp., Enterobacteriaceae, and lactic acid bacteria) begin exponential proliferation, acting as the biological engine that drives the spoilage process.

During storage and transportation, microbial decomposition is the dominant factor driving the decline in meat freshness. In the course of their growth, these microorganisms secrete large quantities of extracellular proteases and lipases. Among these, the proteases systematically degrade myofibrillar and sarcoplasmic proteins in muscle tissue, breaking down macromolecular proteins into peptides and amino acids. These products then undergo further transformations—such as decarboxylation and deamination—yielding an abundance of volatile and malodorous basic nitrogenous compounds, including ammonia, trimethylamine, and dimethylamine. These low-molecular-weight nitrogenous compounds are highly volatile and constitute the principal source of the putrid odors characteristic of spoiled animal-derived foods [31]. The total content of these substances, referred to as total volatile basic nitrogen (TVB-N) and comprising ammonia, dimethylamine, trimethylamine, and related compounds, is regarded as a key indicator for detecting the spoilage of meat and seafood. It also serves as a key chemical index for assessing the degree of meat spoilage, and is specified in relevant national standards such as GB 2707-2016 [32,33]. A pronounced increase in the TVB-N value signifies that proteins have undergone extensive degradation and that the product has entered an unacceptable stage of spoilage.

Concomitant with microbial proliferation and protein degradation, the pH of the meat system exhibits dynamic and regular changes [34]. During the early post-mortem period, anaerobic glycolysis of glycogen yields lactic acid, causing the pH to decline. As storage time progresses, alkaline amines generated from microbial metabolism and protein degradation accumulate continuously, gradually dominating the acid–base equilibrium of the system and driving a trend of rising pH. Hence, pH serves not merely as a parameter characterizing the acidity of the system, but also as a sensitive chemical indicator that comprehensively reflects microbial activity and the progression of protein decomposition.

It can thus be seen that, during meat storage, the total viable count (a microbiological indicator), the TVB-N value (a chemical indicator), and pH (a physicochemical indicator) together constitute a tightly coupled, time-dependent dynamic response network. Microbial proliferation is the causal origin and driving force of spoilage, TVB-N accumulation represents the core chemical manifestation of spoilage, and the rise in pH is an integrated output that reflects the combined influence of the former two on the system environment [35]. This mechanism is schematically summarized in Figure 1.

Figure 1 conceptually illustrates the key stages of meat spoilage, summarizing the transition from microbial proliferation to TVB-N accumulation and the associated pH rise. This schematic framework highlights the sequential “microbiology–chemistry–environment” relationship, which offers a useful reference for identifying potential monitoring targets. pH, functioning as an integrated physicochemical output that reflects the combined influence of microbial proliferation and protein degradation, provides a useful indirect indicator for designing freshness monitoring systems. However, it is crucial to recognize that a pH-responsive indicator film does not directly measure the pH of the meat itself. Rather, the film responds to volatile basic nitrogen compounds (such as ammonia and trimethylamine) that are released from the meat into the package headspace; these compounds dissolve in the hydrated microenvironment of the film and induce a localized pH change within the sensing layer. Therefore, while the meat’s bulk pH trend (initially decreasing then rising) offers a valuable reference for understanding the spoilage chemistry, the actual calibration of indicator film color transitions should be based on headspace volatiles, TVB-N accumulation, microbial counts, and sensory acceptability thresholds under realistic packaging conditions—not merely on the absolute pH value of the meat matrix.

### 2.3. Working Principle of pH-Responsive Indicators and Their General Advantages

Smart packaging enables real-time monitoring of meat freshness by employing pH-responsive indicators. The core mechanism is as follows: during meat storage, volatile organic amines and basic nitrogenous compounds (including ammonia, trimethylamine, and other TVB-N components) are released from the meat into the package headspace. These volatiles diffuse to the indicator film surface, dissolve in the moisture present within the polymer matrix, and release hydroxide ions, thereby raising the local microenvironmental pH of the sensing layer. This localized pH shift is captured by the indicator dye embedded in the film and transduced into a readable color signal [36]. It is important to emphasize that this color change reflects the headspace volatile concentration rather than a direct measurement of the meat’s internal pH; the correlation between the two is indirect and matrix-dependent. Strictly speaking, pH is defined in aqueous solutions (and, by extension, accepted for hydrated solid surfaces); it is not a property of the gaseous headspace itself. The term “pH-responsive” in the context of indicator films refers to the localized pH change within the hydrated sensing layer of the film, which occurs when volatile basic or acidic molecules dissolve in the moisture present in the polymer matrix. This distinction is important: the indicator responds to the dissolution and ionization of volatile compounds in the film’s aqueous micro-environment, not to the gas phase directly.

The color-change logic of pH indicators is thus linked to the food spoilage process through the intermediacy of volatile metabolite accumulation. Foods with high water activity, such as meat and seafood products, are more susceptible to microbial degradation [37]. The pH changes induced in the hydrated sensing layer upon dissolution of headspace volatiles—accompanied by the accumulation of volatile nitrogenous compounds (including ammonia and amines, such as TVB-N)—drive the corresponding color transition on the pH-responsive indicator [38,39]. Consequently, the color response of the film should be calibrated against measured TVB-N levels, microbial counts, and sensory acceptability limits under actual storage conditions, rather than being treated as a general proxy for meat pH. This calibration logic is particularly critical for translating laboratory-scale demonstrations into reliable supply-chain performance.

In practice, the colorimetric response of the indicator film is not governed solely by the concentration of volatile basic nitrogenous compounds. Several extrinsic factors inherent to real-world packaging scenarios can significantly modulate the color-change behavior. For instance, the presence of CO_2_ in modified atmosphere packaging (MAP) is particularly relevant: upon dissolution in the film’s hydrated microenvironment, CO_2_ forms carbonic acid, which can partially neutralize the alkalizing effect of volatile amines and thus shift the apparent color-response threshold. Relative humidity affects both the degree of hydration of the sensing layer and the diffusion rate of hydrophilic volatile amines, while storage temperature governs the volatilization kinetics of spoilage metabolites as well as the reaction rate of the acid–base equilibrium. Additionally, headspace volume and the physical positioning of the film within the package (e.g., proximity to the food surface versus the package headspace) determine the local concentration of volatiles reaching the sensing layer. These factors collectively imply that calibration under simplified laboratory conditions may not be directly transferable to commercial supply-chain applications; rather, the color-response logic should be validated under conditions that closely mimic the actual target packaging system. Moreover, unlike the irreversible biochemical degradation of the food matrix, the acid–base color transition of the indicator is intrinsically reversible—the dye molecule can interconvert between its acidic and basic forms as the local pH fluctuates. However, in a continuously evolving spoilage headspace where volatile amines accumulate progressively, the observed color at any given time represents a dynamic equilibrium rather than a static end-point. This reversibility does not compromise the utility of the indicator for real-time monitoring, but it does necessitate careful interpretation of the color signal, particularly when transient environmental fluctuations (e.g., temporary temperature abuse) may cause reversible color shifts that do not correspond to the actual cumulative quality deterioration.

Based on the deterministic pattern that the end products of meat spoilage lead to a rise in pH, pH-responsive indicators have become the most extensively investigated technical approach in the field of freshness indicators (FI). Their working principle is rooted in fundamental analytical chemistry: the indicator itself is a weak organic acid (HIn) or a weak organic base (InOH), and its conjugate acid and conjugate base forms possess markedly different molecular structures. Consequently, they exhibit distinct absorption spectra in the visible region, manifesting as two sharply contrasting colors.

Taking a typical acidic indicator (such as bromocresol purple) as an example, its color-change process can be simplified as follows [5,36]: HIn (yellow, acidic form) ⇌ H^+^ + In^−^ (purple, basic form). Owing to differences in the conjugated structures of the acidic and basic forms, their absorption spectra undergo a bathochromic or hypsochromic shift, which macroscopically manifests as a color transition. When this indicator is loaded into a porous polymer film and placed inside the package, volatile basic amines (e.g., NH_3_) released during meat spoilage diffuse to the film surface. These amines readily dissolve in the micro-aqueous environment within the film and, through hydration, liberate OH^−^ ions, thereby elevating the pH of the indicator’s local microenvironment. This pH increase drives the above chemical equilibrium to the right, causing the indicator molecules to convert from the acidic form to the basic form, which is macroscopically perceived as a gradual color change in the label. The entire process requires neither a power source nor complex optical instrumentation, realizing a direct transduction of a chemical signal into a visual signal.

The choice of pH indicator for meat freshness monitoring is not arbitrary, but is dictated by the specific chemistry of meat spoilage. Among the sulfonephthalein family of dyes, those with pKa values in the range of approximately 4 to 7—such as bromocresol purple (pKa ≈ 6.03) and bromothymol blue (pKa ≈ 6.72)—are the most commonly employed, whereas indicators with higher pKa values (e.g., m-cresol purple at 8.09, thymol blue at 8.53) are rarely used. This is not coincidental. During meat spoilage, the pH of the matrix never reaches strongly basic values, even when protein catabolism is well advanced, because the muscle tissue is a buffered system. Moreover, the volatile basic nitrogen compounds that are eventually released into the headspace—principally ammonia and biogenic amines—exist predominantly in their protonated (positively charged) form within the acidic to neutral matrix and cannot easily partition into the gas phase unless they are in their neutral (deprotonated) state. This thermodynamic constraint, combined with the limited pH rise in the hydrated sensing layer upon dissolution of headspace volatiles, restricts the effective color-change window to the near-neutral to mildly alkaline region. Consequently, indicators with pKa values in the 6–7 range offer the best compromise between sensitivity to volatile amine accumulation and practical response under real-world spoilage conditions. This fundamental acid–base and partitioning chemistry, while often overlooked in the applied food science literature, directly explains the empirical choice of dyes observed in published studies [40,41].

It is worth noting that the design rationale for freshness indicators is not unique. Redox indicators (such as methylene blue) can be reduced and thus change color by reducing substances (e.g., NADH) generated from microbial metabolism; however, they are susceptible to interference from other reducing components present in the food matrix, which limits their scope of application. Enzyme-based indicators, in contrast, achieve high specificity by immobilizing specific oxidases (e.g., amine oxidase) that selectively catalyze reactions with target spoilage compounds. Nevertheless, the activity and stability of enzymes are extremely sensitive to environmental factors such as temperature and pH, and the complexity of system construction and high costs hinder their large-scale application [42,43,44,45]. By comparison, pH-responsive indicators exhibit irreplaceable general advantages: their principle is simple, centering solely on acid–base equilibrium without the need for complex bio-recognition elements; the system is stable and not readily affected by non-specific interferences from the complex food matrix; and the cost is controllable, with simple preparation, making them easy to popularize. It is precisely this directness, robustness, and economic viability in principle that positions them as the most feasible pathway for translating laboratory research into practical application. This also points to clear optimization directions for subsequent research: building upon the inherent advantages of their principle, challenges concerning safety and environmental friendliness that they currently face should be addressed through material innovation, such as the use of natural dyes.

Enzymatic recognition elements, exemplified by amine oxidase, offer specificity for biogenic amine detection [42,43,44], but their susceptibility to environmental variations and limited long-term stability hinder their direct integration into food packaging systems [45].

In contrast, for meat products in which volatile basic nitrogenous compounds serve as the hallmark of spoilage, pH-responsive indicators exhibit irreplaceable general advantages. First, target generality: alkaline amines are common end products of protein degradation driven by diverse microorganisms and are not restricted to any single bacterial species. Second, direct correlation: pH changes are highly correlated with TVB-N accumulation and the degree of sensory deterioration, endowing the indicator results with clear significance. Third, system stability: at its core lies a simple acid–base equilibrium, rendering it relatively robust to non-specific interferences from the complex food matrix; moreover, preparation is straightforward and the cost remains controllable. It is precisely this directness, robustness, and economic viability in principle that positions these indicators as the most feasible pathway for translating laboratory research into practical applications. This also points to clear optimization directions for subsequent research: building upon the inherent advantages of their working principle, challenges concerning safety and environmental friendliness that they currently face should be addressed through material innovation, such as the use of natural dyes.

## 3. Comprehensive Evaluation Model for Food Freshness: The Theoretical Cornerstone of Intelligent Indicator Film Design

### 3.1. From Single Indicators to Comprehensive Assessment: The Necessity of Establishing an Accurate Monitoring Framework

The core function of an intelligent indicator film is to accurately and promptly reflect critical changes in food quality. A fundamental question thus arises: with what “spoilage signal” should the color transition point of the film be synchronized? If this signal is simplistically equated to the absolute value of a single chemical indicator (e.g., pH), one may fall into a monitoring pitfall. As discussed previously, meat spoilage is a dynamic process driven by the interactions among microorganisms, biochemical reactions, and the food matrix. The deterioration of meat quality manifests as the synergistic outcome of multiple Key Quality Indicators (KQIs), such as total viable count (TVC), total volatile basic nitrogen (TVB-N), and pH value.

In published studies, most colorimetric food freshness indicators based on natural pigments rely solely on color change observed before and after quality evaluation, with insufficient investigation into the correspondence between this color change and the actual spoilage process. To further enhance monitoring reliability, incorporating multiple indicators to verify freshness has emerged as a new research trend [46].

For instance, Hashim et al. [20] validated the freshness monitoring performance of their colorimetric film by measuring changes in TVB-N values of shrimp during storage at 25 °C. Several studies have examined the variation patterns of TVB-N, pH, drip loss, and color difference (ΔE) in minced pork during storage, and have attempted to establish a linear relationship between pH and TVB-N for indirect freshness estimation.

However, the aforementioned approaches still largely rely on single indicators or a simple enumeration of multiple individual parameters to assess the freshness of meat products. Although changes in these indicators are intrinsically correlated, they are not synchronized, and their sensitivities to internal and external factors differ significantly. For example, the initial microbial composition of the meat or transient temperature fluctuations may cause abnormal variations in metabolites derived from specific microorganisms (such as certain organic acids), leading to short-term fluctuations in pH. At this point, the total viable count and TVB-N may not have changed significantly, and sensory indicators may not yet have reached the spoilage threshold. Conversely, under certain modified atmosphere packaging conditions, microbial growth is inhibited and TVB-N accumulates slowly, yet lipid oxidation may be intensified. In such cases, relying solely on a single microbial or TVB-N indicator cannot fully reflect the quality deterioration that has already occurred. This indicates that judging freshness based on any single KQI is inherently haphazard and one-sided, potentially resulting in the dual risks of “false negatives” (spoilage goes undetected) and “false positives” (acceptable products are mistakenly discarded). The fundamental cause of this risk is that a single indicator cannot capture the complexity of the spoilage process.

It is precisely based on this recognition that the introduction of multivariate statistical methods for the comprehensive analysis of multiple quality attribute indicators has become an effective approach to overcoming the one-sidedness of information derived from individual indicators. Through methods such as Principal Component Analysis (PCA), Hierarchical Cluster Analysis (HCA), and Fisher Discriminant Analysis (LDA), multiple quality attribute indicators related to freshness can be comprehensively analyzed, thereby enabling a more robust overall judgment of meat freshness and addressing the problem of one-sided and insufficient information from individual quality indicators [20]. Lin et al. investigated chilled beef during cold storage and, by combining PCA, HCA, and LDA with sensory scores and color changes, successfully constructed a freshness evaluation model that validated the feasibility of this multivariate approach [47].

However, it is important to note that while such multivariate statistical frameworks have proven effective for quality grading, their direct application to the calibration of indicator film color-response thresholds—i.e., using the model-derived critical spoilage state to define the film’s decisive color transition point—has not yet been explicitly demonstrated in the literature. Most published studies on natural pigment-based indicator films still rely on empirical correlation between color change and individual spoilage indicators such as TVB-N or pH, rather than on a statistically validated multi-indicator comprehensive state [19,20,25].

This recognition does not diminish the value of the PCA–CA–DA framework; rather, it highlights its potential as a standardized design protocol for future indicator film development. By applying this framework to calibrate the color-transition logic against a rigorously defined “comprehensive spoilage state,” the inherent subjectivity and one-sidedness of current empirical approaches can be overcome. The establishment of such an evaluation system provides the objective basis that should be followed—rather than relying on subjective conjecture—in addressing the question of “when the film should change color.”

### 3.2. Application of Multivariate Statistical Methods in Deciphering Spoilage Processes and Defining Quality Grades

To construct the comprehensive evaluation system described above, multivariate statistical methods serve as the core tool. Their principal value lies in transforming high-dimensional, coupled key quality indicator (KQIs) data into intuitive information that clearly characterizes spoilage stages and quality grades, thereby providing a useful quantitative framework for the design of indicator films. This transformation process generally follows a logical chain of ‘source tracing–classification–discrimination’ (Figure 2 provides a schematic overview of this analytical pipeline).

These three steps constitute a logically progressive and complete analytical chain: PCA aims to identify the principal contributing factors of spoilage, CA is responsible for establishing objective quality classification criteria, and DA is dedicated to developing a generalizable grade discrimination model. Advancing layer by layer, the three methods ultimately provide a useful quantitative framework for the color response thresholds of indicator films. The following sections will elaborate on the core functions and specific applications of each method.

First, principal component analysis (PCA) plays the role of a “data prospector.” As a widely used dimensionality reduction and data exploration technique, it is of significant value in identifying the main features or patterns within a dataset [48]. Its working principle is to select the largest eigenvalues and their corresponding eigenvectors as principal components, capturing the most prominent variance information in the data and thereby revealing underlying pattern relationships and key characteristics. This technique not only aids in understanding the intrinsic structure of the data but also supports data visualization, classification, clustering, and other analytical tasks [49].

In the context of freshness evaluation, PCA transforms multiple correlated variables—such as total viable count (TVC), TVB-N values, and pH—into a small number of mutually independent principal components that retain most of the information of the original data through linear transformation. This transformation is made possible because of the multicollinearity among the original variables, which PCA eliminates via orthogonal transformation, thereby removing such redundancy. Typically, the first principal component captures the differences among samples to the greatest extent, and its scores are often highly correlated with storage time, thus serving as a “comprehensive spoilage progress index.” By analyzing the contributions (loadings) of each original variable to the principal components, it is possible to identify which indicators (e.g., TVB-N or certain characteristic spoilage microorganisms) show the strongest association with the principal spoilage-related variance. However, it should be emphasized that PCA reveals patterns of co-variation and does not by itself establish causal relationships; the interpretation of loading patterns should always be grounded in independent microbiological and biochemical knowledge of the spoilage system.

This capability of PCA has been demonstrated in studies on TVB-N prediction, where the integration of multi-source sensing data—such as hyperspectral imaging and colorimetric sensor arrays—has been explored to improve prediction accuracy [50,51,52]. For instance, researchers have employed backpropagation artificial neural networks (BPANN) to capture the complex nonlinear mapping between TVB-N content and multi-source data, with the number of principal components optimized via cross-validation to establish robust prediction models [50,52]. Studies have confirmed that fusing two independent sensing systems—hyperspectral imaging (optical sensor) and a colorimetric sensor-based olfactory/electronic nose system (odor sensor)—can significantly enhance TVB-N prediction performance, as the multi-source data fusion complements the respective information blind spots of each system and forms a more complete representation of quality characteristics [49].

However, it should be noted that these studies focus on predicting TVB-N content as an analytical goal, rather than on calibrating indicator film color-response thresholds against a multi-indicator comprehensive spoilage state. The direct translation of such PCA-based prediction models into a design parameter for pH-responsive indicator films—i.e., defining the exact pH or volatile concentration at which the film should undergo a decisive color change—remains an open challenge. This gap further underscores the need for the PCA–CA–DA workflow proposed in this review as a systematic approach to bridge the analytical prediction of spoilage markers and the engineering design of indicator films.

Once the dominant drivers of spoilage have been identified, it is necessary to objectively classify quality categories with practical management significance, such as “fresh,” “sub-fresh,” and “spoiled.” At this stage, cluster analysis (CA) plays a critical role. CA performs automatic classification based on all KQIs data, grouping samples according to their overall similarity. For example, CA may cluster samples from the early storage period into one group (fresh), those from the middle period into another (sub-fresh), and those from the late period into a third (spoiled). This process, proceeding from the intrinsic structure of the data, objectively defines the multi-indicator numerical ranges corresponding to each freshness grade, thereby avoiding the subjectivity inherent in manual classification and providing a scientific basis for the color zoning design of indicator films. In practice, the automatic grouping produced by CA does not automatically translate into predefined quality grades such as “fresh,” “sub-fresh,” or “spoiled.” The assignment of cluster labels requires external anchoring against independent reference criteria—including microbiological counts (e.g., TVC), chemical indices (e.g., TVB-N), and sensory evaluation scores—to ensure that the clusters correspond to meaningful and actionable freshness categories. Furthermore, the reliability of CA results is contingent on adequate sample size, balanced class representation across storage time points, and the use of appropriate validation procedures (e.g., cross-validation) to guard against overfitting and to assess the stability of the clustering structure.

However, PCA and CA are primarily used to describe the structure of known data and cannot be directly applied to the determination of new samples. To achieve automatic discrimination of new samples, a predictive model needs to be established. Discriminant analysis (DA) uses the categories defined by CA as the training set to construct discriminant functions. When the measured values of various KQIs of an unknown sample are input, the DA model can calculate the probability of its belonging to each category, thereby enabling rapid and objective grade determination. For example, one study utilized near-infrared hyperspectral imaging to classify cheeses of different brands and thoroughly investigated the overfitting issues and generalization ability of models such as partial least squares discriminant analysis (PLSDA) and linear discriminant analysis (LDA), in order to reduce the risk of overfitting and achieve accurate discrimination of unknown samples [53].

A properly validated DA model—with independent test sets and rigorous cross-validation—can provide a quantifiably verifiable reference for the accuracy of indicator film color interpretation. Specifically, the color-change outcomes of the film should be evaluated against the DA-assigned quality grades of the same samples. This consistency check transforms the assessment of film performance from a subjective judgment of “whether the color looks like spoilage” to a more objective evaluation of “whether the classification agrees with a statistically validated reference.” However, it is important to note that the generalizability of the DA model depends on the representativeness of the training set; factors such as sample size, category balance, and the independence of validation datasets must be carefully addressed to avoid over-optimistic performance claims. Thereby providing a scientific performance evaluation framework for the practical application of intelligent indicator films.

The above analysis demonstrates that PCA, CA, and DA are not isolated data processing techniques. Their interconnection and synergistic operation together constitute a complete methodological framework, spanning from the analysis of spoilage patterns to quality grade determination. The effectiveness of this framework is built upon a progressive logic of ‘dimensionality reduction simplifies cognition, clustering defines knowledge, and discrimination extends application’ as detailed in the step-by-step flowchart shown in Figure 3.

In summary, through the modeling workflow of “PCA source tracing–CA classification–DA discrimination,” the ultimate output of the research is a set of quantitative freshness evaluation criteria tailored to a specific food-storage system. The physical core of this set of criteria lies in identifying the critical spoilage threshold that marks the transition from quantitative changes to qualitative change. It directly addresses the core parameter question in the design: the color transition point of the indicator film must precisely correspond to the aforementioned critical spoilage state. For pH-responsive films, this means that the pH response point (or narrow interval) at which a decisive color change occurs must be set near the critical pH value determined by the model. In this way, the complex process of food spoilage is condensed into a concise, engineerable design parameter, achieving a seamless transition from the theoretical model to material design.

## 4. Core Materials for Intelligent Indicator Films: The Shift from Synthetic to Natural Dyes

### 4.1. Fundamentals of the Chromogenic Chemistry of pH Indicators

The key to achieving visualization in intelligent indicator films lies in the specific response of the dye molecules embedded within the film to hydrogen ion concentration, a process governed by well-defined physicochemical principles. In essence, the conjugated π-electron system of the dye molecule (typically an organic weak acid, HIn, or a weak base) undergoes a reversible structural interconversion in response to ambient pH changes, which shifts its maximum absorption spectrum and macroscopically manifests as a color change. The shift in this equilibrium (HIn ⇌ H^+^ + In^−^) is essentially the dynamic variation in the proportion of the two molecular species—the acid-color form and the base-color form. Owing to differences in chemical bonding, electron density distribution, and spatial configuration, the two forms exhibit distinct frontier molecular orbital energy gaps and therefore display markedly different photon absorption characteristics. Understanding this fundamental principle is the logical starting point for screening and designing any pH-responsive indicator material, as it determines the material’s intrinsic response properties, such as the color-change point (pKa) and color contrast.

Taking natural dyes as an example, their precise “structure–pH–color” correlation provides a direct basis for material selection. Anthocyanins are among the most extensively studied natural pH indicators. They are rich in phenolic compounds, which impart bright colors to fruits and vegetables, and undergo pronounced structural changes in response to varying pH conditions [54,55]. These pigments are widely present in the vacuoles of plants such as roses, grape skins, and red cabbage, and can be obtained as raw anthocyanin extracts through suitable extraction processes [56,57]. Their chromogenic mechanism originates from the stepwise rearrangement of the pyran ring (C-ring) with changing pH: under strongly acidic conditions (pH < 3), the stable flavylium cation structure renders a red color; as the pH rises to 4–5, nucleophilic addition of a water molecule yields the colorless carbinol pseudobase; and in near-neutral environments, deprotonation generates a bluish-purple quinoidal base. The color intensity and stability of anthocyanins are influenced by multiple factors, including the molecular structure of the dye, pH, temperature, light, as well as enzymes, oxygen, sugars, and their metabolites [58].

The color-change mechanism of betacyanins (a subclass of betalains, which also includes yellow betaxanthins) differs from that of anthocyanins. Betacyanins retain a reddish-purple hue over the acidic to neutral pH range of 3–7; however, once the environment becomes alkaline (pH > 7), the chromophore undergoes not only deprotonation of phenolic hydroxyl groups—which disrupts the conjugated system—but also potential hydrolytic cleavage and irreversible degradation, collectively leading to a pronounced fading to yellow or brownish hues. This irreversible behavior contrasts with the reversible acid–base equilibrium observed for anthocyanins and should be carefully considered when designing indicator films for monitoring alkaline spoilage environments.

These molecularly determined pH response windows with well-defined boundaries imply that, when developing indicator films for specific food systems, one must first select dyes from the natural pigment repertoire whose color-change points match the target range, or anticipate the necessary adjustments of their response intervals through materials science approaches. A deep understanding of the chromogenic mechanism, therefore, serves as the theoretical foundation for all subsequent material optimization and engineering design.

### 4.2. Performance Advantages and Safety Risks of Synthetic Dyes: The Inevitability of a Paradigm Shift

However, single-dye solutions suffer from certain drawbacks, such as a narrow pH color-change range and inconspicuous color transitions [59]. A growing body of research has found that pH indicator labels prepared by blending multiple dyes can achieve superior indicator performance [60].

In the early stages of intelligent packaging technology development, a series of synthetic organic dyes—such as methyl red, bromocresol purple, and bromothymol blue—became mainstream choices owing to their excellent and standardized photochemical properties. From a purely technical standpoint, these dyes offer significant advantages: first, they exhibit exceptionally high color contrast, with the acid and base forms typically being complementary colors, enabling intuitive visual interpretation; second, they demonstrate outstanding response sensitivity, accomplishing a sharp color transition within a narrow pH interval; third, they possess a broadly tunable pKa range, capable of meeting the demands of various monitoring windows. From the perspective of indicator functionality, synthetic dyes exhibit near-ideal performance.

However, when the evaluation perspective shifts from laboratory functionality validation to real-world application scenarios in food packaging, the inherent drawbacks of synthetic dyes constitute insurmountable obstacles. The core contradiction lies in the antagonism between their excellent indicator performance and unacceptable biosafety and environmental risks. The vast majority of synthetic dyes are derived from benzene-series, anthraquinone, or azo petrochemical feedstocks, and their molecular structures, synthetic intermediates, or metabolites may possess potential cytotoxicity or genotoxicity. In the complex environment of food packaging, especially in the presence of moisture, fats, or organic acids, there is a definite risk of these small dye molecules migrating from the polymer matrix into the food, which directly contravenes the overriding principle of food packaging—safeguarding consumer safety. Studies have indicated that when synthetic dyes are applied in food safety testing, food may be contaminated by the volatilization of toxic substances, thereby jeopardizing human health [61]. For these reasons, chemical dyes are not ideal materials for the production of intelligent food packaging [62,63]. Furthermore, the production processes of synthetic dyes are characterized by high energy consumption and heavy pollution, and the dyes themselves are poorly biodegradable, resulting in a substantial environmental burden in the life-cycle assessment of packaging waste. This runs counter to the global development strategies of a circular economy and green packaging.

Therefore, although synthetic dyes historically facilitated the initial realization of the intelligent packaging concept, their deficiencies in the two fundamental dimensions of food safety and environmental sustainability determine that they cannot serve as the cornerstone for next-generation intelligent packaging materials with large-scale commercial potential. This profound contradiction has compelled a fundamental paradigm shift in research, driving the innovation focus of the field toward naturally derived material systems that combine functionality with safety and eco-friendliness.

Against this backdrop of transformation, research on natural pigments has become a hot topic. Fan et al. [19] evaluated the performance of anthocyanins in preservation and intelligent indication by incorporating them as a pH indicator into intelligent active packaging films based on chitosan and pectin. They investigated the effect of different dosages of AEBR on the physical properties of the composite films, and characterized the changes in AEBR and AEBR-loaded pectin/chitosan films under a volatile ammonia environment and a meat preservation model using UV–vis spectroscopy, in order to explore the practical application feasibility and the color-change mechanism of anthocyanin-based films.

Furthermore, pigment blending is also an effective strategy for broadening the color-change range of natural pigment-based indicators [23]. Different pigments possess distinct colorimetric endpoints, and the color superposition produced by blending can create and present new hues, which themselves shift with changing pH. Consequently, the color-change range is widened and the sensitivity of natural pigment-based indicators is enhanced. For instance, Chen et al. [64] premixed curcumin and anthocyanins and incorporated them into a film matrix, and found that during chicken storage, the pH increase caused by the accumulation of volatile nitrogenous compounds resulted in more pronounced color changes in the film containing the mixed pigments than in the single-pigment film, confirming the improvement effect of the blending strategy on pH response characteristics. Based on a similar approach, Rhim’s group developed a functional pH-responsive color-changing film based on a mixture of anthocyanins and shikonin; by adjusting the ratio of the two pigments, they successfully broadened the film’s color-change range under different pH conditions [22].

While natural dyes offer a clear advantage in terms of biocompatibility, it is essential to avoid the assumption that “natural” equates to “entirely risk-free” regarding potential contamination of food. In practice, when natural dyes are loaded into metal–organic frameworks (MOFs) or encapsulated within nanoparticles, the possible migration of inorganic metal ions (e.g., Zr^4+^ from UiO-66, Zn^2+^ from ZIF-8), organic ligands, or the carrier materials themselves into the food matrix cannot be overlooked. Similarly, although chemical crosslinking agents such as glutaraldehyde are effective in enhancing mechanical properties and reducing dye leaching, their well-documented cytotoxicity raises legitimate safety concerns when used in food-contact materials; safer alternatives such as genipin should be prioritized wherever feasible. Furthermore, the degradation products of natural dyes—resulting from oxidative, photochemical, or thermal reactions during storage—may themselves exhibit unknown toxicity profiles. It is important to emphasize that regulatory safety assessments for food-contact materials are typically conducted on the final assembled device under realistic migration conditions, rather than on isolated dye molecules or intermediate components alone. For synthetic dyes such as sulfonephthaleins, studies have shown that the actual amount migrating to food from a properly formulated label can fall within acceptable safety limits when the device is tested as a whole, even if the dye itself exhibits toxicity at higher concentrations. This distinction is crucial: the absence of commercial adoption of synthetic-dye-based indicators cannot be attributed solely to inherent dye toxicity, as migration levels in the final device may be negligible. Rather, the shift toward natural dyes is driven by a combination of consumer preference, regulatory precaution, and marketing considerations that extend beyond strict toxicological necessity. Nevertheless, for MOF-based systems, the presence of metal ions introduces a distinct and more stringent regulatory challenge, as any metal-containing substance is subject to much stricter migration limits under regulations such as EU No 10/2011. Therefore, a comprehensive risk assessment that covers the entire material system, including dyes, carriers, crosslinkers, and their potential degradation byproducts, is imperative for the responsible design and regulatory acceptance of commercial-scale intelligent packaging.

### 4.3. Functional Enhancement Strategies for Natural Dyes: From Laboratory Research to Practical Application

Although chemically synthesized pigments offer advantages such as high stability, ease of production, and cost-effectiveness, safety remains the paramount consideration in food applications. The potential toxicity risks associated with synthetic pigments [64] have drawn increasing skepticism against the backdrop of rising food safety concerns. This latent hazard further highlights the development prospects of natural dyes. By reducing safety risks, natural dyes also address consumer demands for food safety. Moreover, many natural dyes inherently possess biological activities such as antioxidant and antimicrobial properties [65], enabling them to indicate food freshness while simultaneously retarding quality deterioration to a certain degree, thereby achieving synergistic enhancement of indicator function and bioactivity. Taking anthocyanins as an example, as natural antioxidants, their reducing properties can inhibit the oxidation of other molecules, thus serving the dual functions of preservation and monitoring [58].

Natural pigments represented by anthocyanins, betalains, and curcumin, with their inherent biocompatibility, edible safety, renewable feedstocks, and environmental friendliness, offer a compelling response to the safety concerns surrounding synthetic dyes and have thus become an ideal starting point for innovation in smart packaging materials. However, when natural extracts are directly incorporated into functional packaging components—particularly into freshness indicator films, which demand stringent long-term stability and reliability—their “natural” attributes also give rise to a series of formidable technical challenges: narrow and fixed color-change ranges governed by pKa, chemical instability arising from sensitivity to environmental factors such as light, heat, and oxygen, high proneness to migration due to strong water solubility, and poor compatibility with common polymer film-forming matrices. If these drawbacks are not effectively addressed, natural dye-based indicator films will remain confined to laboratory demonstrations, failing to meet the shelf-life and operational reliability requirements of real commercial products. Consequently, the current frontier and core focus of research in this field has shifted comprehensively from early-stage feasibility validation to a new phase of “functional enhancement” of natural dyes through systematic materials science and engineering strategies.

To provide a structured overview of the material systems discussed in this section, Table 2 summarizes the pigment sources, film matrices, and fabrication strategies reported for representative pH-responsive indicator films.

#### 4.3.1. Stabilization and Migration Control of Natural Dyes via Micro/Nano-Encapsulation Technology

Micro/nano-encapsulation is a strategy that provides physical protection for fragile dye molecules. This technique employs biodegradable materials—such as modified starch nanoparticles, chitosan nanoparticles, zein, or liposomes—as wall materials to encapsulate natural dyes, forming core–shell structured microcapsules or nanoparticles. A schematic illustration of this structure is presented in Figure 4.

This “outer-wall-inner-core” topological architecture serves as the physical foundation for all subsequent performance enhancements. Compositing or encapsulating two or more materials at the nanoscale enables the construction of higher-level nanostructured assemblies, imparting multiple new properties that cannot be achieved by single nanoparticles and thereby expanding their application boundaries [66]. For instance, Yamdej et al. [67] demonstrated that mulberry anthocyanin adsorbed onto calcium alginate microspheres exhibited a markedly higher retention rate than unencapsulated samples after treatment at 80 °C for 10 h. These examples substantiate the notable effectiveness of micro/nano-encapsulation strategies in enhancing the stability and protective properties of natural dyes.

The reinforcement mechanism of this strategy is manifested primarily in two aspects. First, the dense coating layer effectively blocks external factors such as ultraviolet radiation, oxygen, and water vapor that induce photodegradation, oxidative degradation, and hydrolysis of the dyes, thereby substantially enhancing the stability of the dyes during processing, storage, and application. Research data indicate that the color retention rate of nano-starch-encapsulated anthocyanins after accelerated light and thermal aging tests can be several times higher than that of the unencapsulated group. Second, the coating layer functions as a diffusion-controlling barrier that finely modulates the kinetic process of dye molecule migration into the external environment. By adjusting the thickness, density, and hydrophilicity/hydrophobicity of the wall material, a programmable design of the dye release rate can be achieved, thereby effectively addressing the challenge of rapid leaching and migration of natural dyes from hydrophilic film matrices and ensuring the durability and safety of the indicator function. This strategy is not only applicable to indicators but also provides a general technical platform for constructing active packaging systems with sustained-release functionality.

#### 4.3.2. Application of Porous Framework Materials in the Immobilization of Natural Dyes

Novel porous crystalline materials, represented by metal–organic frameworks (MOFs) and covalent organic frameworks (COFs), have provided a revolutionary solution for the immobilization of natural dyes. MOFs are a class of three-dimensional porous crystalline materials constructed from inorganic metal ions or clusters and organic ligands containing nitrogen or oxygen atoms through coordination bonds [68]. Owing to their exceptionally high porosity, specific surface area, and regular nanochannels with tunable pore sizes, MOFs have found extensive applications in gas separation and storage, biomolecular encapsulation, energy, and other fields [69]. As illustrated in Figure 5, their microstructure features regular nanochannels, which provide an ideal spatial confinement environment for the stable encapsulation of dye molecules.

In the immobilization of natural dyes, the regular nanochannels of MOFs can effectively suppress the aggregation and oxidation of dye molecules through the confinement effect, while simultaneously concentrating spoilage markers within the channels via the enrichment effect, thereby synergistically enhancing the response sensitivity and signal intensity of the indicator film. In contrast, COFs are constructed from light elements connected by covalent bonds; although they possess a lower density, they exhibit even more prominent chemical stability.

Loading natural dyes into the nanochannels of MOFs (e.g., UiO-66 and ZIF-8) via impregnation, in situ synthesis, or other strategies can generate multiple synergistic reinforcement effects. Taking the UiO-66 series as an example, this material has attracted considerable attention for its exceptional thermal, chemical, and mechanical stability, and its selective recognition capability and detection sensitivity toward specific gases can be further enhanced by introducing functional groups such as -Br. Studies have demonstrated that novel indicator films can be fabricated by loading anthocyanins onto UiO-66 and UiO-66-Br carriers, followed by compositing with polyvinyl alcohol (PVA) and chitosan (CS). Over a pH range of 2–12, the film was observed to undergo a gradual color transition from red to blue and eventually to yellow. The investigation revealed that the molecular interactions between anthocyanins and MOFs suppressed dye migration within the indicator film. Concurrently, the porous architecture of MOFs increases the specific surface area available for gas contact, thereby enhancing the color sensitivity of the indicator film [24].

The underlying reinforcement mechanisms can be summarized into two aspects. First, the potent nano-spatial confinement effect firmly immobilizes dye molecules within the MOF nanochannels, physically and substantially restricting their migration and leakage under typical processing and storage conditions. Second, the porous architecture of MOFs increases the specific surface area available for gas contact, thereby enhancing the color sensitivity of the indicator film. Both effects have been confirmed in the context of anthocyanin-loaded UiO-66 systems, as discussed above [24].

More importantly, for some elaborately designed MOFs (e.g., UiO-66-NH_2_ constructed with 2-aminoterephthalic acid ligands), the basic functional groups decorating the surface of their nanochannels possess specific chemisorption toward volatile alkaline amine gases (such as ammonia and trimethylamine) generated during meat spoilage. Integrating pH-responsive dyes with such MOFs enables the construction of a cascade response mechanism of “selective adsorptive enrichment–in situ micro-environmental pH alteration–rapid dye color development.” This synergistic effect not only markedly enhances the detection sensitivity of the indicator film toward low-concentration spoilage gases (i.e., lowering the limit of detection), but also strengthens its anti-interference capability against the complex background of food volatiles (i.e., improving selectivity), representing a significant evolution of indicator materials from passive response to active recognition and signal amplification.

#### 4.3.3. Application of Blending Strategies and Matrix Engineering in the Systematic Optimization of Indicator Film Performance

Dye blending represents one effective strategy to overcome the limitations of single dyes and achieve customized visual signals.

After optimization at the dye level, systematic design from the perspective of the film-forming matrix is further required to comprehensively enhance the overall performance of indicator films. Currently, the development of packaging materials based on natural polysaccharides has become a research hotspot, as these polysaccharides are highly valued for their renewability, biodegradability, non-toxicity, edibility, and sustainability [70,71]. Polysaccharide matrices commonly used in food packaging include cellulose, starch, chitosan, alginate, and carrageenan [72,73,74].

Blending strategies represent an effective approach to overcoming the limited color-change range of single dyes and achieving customized design of visual signals. By physically blending or chemically coupling two or more natural dyes that possess different pKa values and complementary color-change sequences—such as anthocyanins, which exhibit pronounced color variations across a broad pH range (from red in acidic conditions to blue/purple in neutral and yellow in alkaline), and curcumin, which transitions from yellow to orange/reddish in the weakly alkaline region (approximately pH 7.5–9.0) while showing a more subdued response under acidic conditions—it is possible to artificially “splice” together a continuous color-changing band that spans a wider pH range, or to construct a “stepped” indicator pattern with multiple color transition points. This enables the indicator film to distinguish different quality states—such as fresh, sub-fresh, and spoiled—with greater precision, thereby providing richer visual information.

To counteract the inherent chemical instability of anthocyanins under environmental stress, encapsulation within protein–polysaccharide carrier systems has proven effective in substantially improving their retention and functional stability during storage [75,76,77].

At the film level, systematic optimization of the film-forming matrix—referred to as matrix engineering—is a critical step in determining the ultimate practical performance of indicator films.

At the macroscopic film level, the systematic optimization of the film-forming matrix is a critical step in determining the ultimate practical performance of indicator films. This optimization involves multiple dimensions: first, selecting a polymer that offers a balance of mechanical properties, barrier properties, and biocompatibility as the base matrix. Studies have demonstrated that combining chitosan with polyvinyl alcohol (PVA) can improve film-forming properties. Koosha et al. [21] used chitosan and PVA as substrates, loaded with black carrot anthocyanins, to prepare a smart nanocomposite film, in which the addition of clay further reduced the water vapor permeability of the film and enhanced its antibacterial properties. Similarly, Merz et al. [78] developed an indicator film based on chitosan, PVA, and anthocyanins for monitoring shrimp freshness, confirming that the components can achieve effective dispersion and tight integration within the film matrix.

Secondly, the matrix can be modified by introducing functional additives. Commonly employed modification strategies include: incorporating plasticizers such as glycerol to improve the film’s flexibility and adhesion while preventing embrittlement; compounding with nano-reinforcing phases such as cellulose nanocrystals and montmorillonite to substantially enhance the tensile strength, elastic modulus, and barrier properties against water vapor and oxygen; and subjecting the matrix molecules to chemical crosslinking (e.g., using genipin or glutaraldehyde) or hydrophobic modification (e.g., grafting with long-chain alkyl groups) to strengthen the polymer network and reduce the film’s water absorption and swelling, thereby immobilizing dye molecules at the microscale while improving the film’s moisture resistance and durability at the macroscale. Liu et al. [25] developed a smart packaging film combining hydroxypropyl methylcellulose (HPMC), salicylic acid, and curcumin, which not only exhibits pH-responsive color-changing functionality but also demonstrates good thermal stability and antibacterial activity. After applying it to the packaging of pork and shrimp, the researchers thoroughly examined the correlation between the film’s color changes and spoilage indicators such as TVB-N values and pH values, verifying its monitoring efficacy in practical scenarios.

A comprehensively optimized composite film system should achieve an optimal balance among stable immobilization of the dye, efficient diffusion of the target gas, mechanical reliability of the film itself, and overall processing feasibility. This systematic optimization strategy, spanning from dye to matrix, represents precisely the key to transitioning indicator films from laboratory prototypes to practical applications.

The development of core materials for smart indicator films has evolved from the direct application of natural substances, through targeted functional enhancement to address their inherent limitations, such as instability, and is now progressing toward the integrated design of multi-component systems. The safety and eco-friendly nature of natural dyes remain their irreplaceable foundation, while strategies such as micro-/nano-encapsulation for enhancing stability, porous frameworks for achieving immobilization, and blending chemistry and matrix engineering collectively provide the key technical support for practical application. At present, the research focus has converged on the construction of multifunctional integrated systems, striving to simultaneously overcome multiple challenges—including sensitivity, stability, resistance to migration, and mechanical durability—through synergistic design among the components.

While Table 2 summarizes the material composition of representative systems, Table 3 further compiles their reported performance characteristics—including response behavior, stability, and migration status—to facilitate a comparative assessment of their practical applicability.

Comparative assessment of functional enhancement strategies. Based on the literature surveyed in this review, the three principal strategies for enhancing natural dye performance—micro/nano-encapsulation, MOF immobilization, and pigment blending—offer distinct advantages and face different challenges when considered for practical implementation.

Micro/nanoencapsulation currently appears to be the most technically mature and versatile approach, as it provides effective protection against environmental degradation, offers tunable release kinetics, and can be implemented using generally recognized as safe (GRAS) wall materials. Its major barriers to scale-up are the complexity of preparation and the potential for increased production costs. Nevertheless, for short- to medium-shelf-life products where cost sensitivity is moderate, encapsulation-based indicators represent the most practical near-term solution.

MOF immobilization, while demonstrating exceptional performance in terms of dye stability, sensitivity, and anti-interference, faces more substantial hurdles for commercialization. The relatively high cost of MOF synthesis, the as-yet unresolved concerns regarding the potential migration of metal ions and organic linkers, and the limited availability of food-grade MOFs all constrain their immediate industrial applicability. In the longer term, however, MOFs—particularly those derived from biocompatible metals and ligands—hold the greatest potential for achieving a step-change improvement in indicator performance, provided that safety and cost issues can be adequately addressed.

Pigment blending offers the simplest and most cost-effective route to broadening the color-change range and achieving multi-stage freshness discrimination. Its primary limitation lies in the inherent instability of certain natural pigments (notably anthocyanins) under real-world storage conditions, which can compromise the reliability of the blended system over extended periods. For applications where cost and simplicity are the overriding priorities, blending strategies remain an attractive option, especially when combined with mild stabilization measures.

In summary, we consider micro/nano-encapsulation to be the most immediately promising strategy for practical application, owing to its balance of effectiveness, safety, and scalability; pigment blending offers a low-cost complementary approach for specific use cases; and MOF immobilization, despite its superior technical performance, requires further safety validation and cost reduction before it can be deployed in commercial packaging systems. This comparative assessment is intended to guide future research toward the strategies most likely to achieve real-world impact.

## 5. Summary and Outlook

Real-time monitoring of food quality is a crucial link in ensuring food safety and reducing waste. Faced with the dual challenges of global food waste and food safety assurance, the development of real-time and intelligent quality monitoring technologies has become an urgent demand in the field of food packaging. This review summarizes the research progress on smart packaging materials for real-time food quality monitoring, with a particular focus on pH-responsive freshness indicator films, and reveals that this field is undergoing a profound transformation from empirical exploration to rational design.

At the heart of this transformation lies the synergistic development of three mutually reinforcing technological pathways: first, theoretical modeling based on the biochemical processes of meat spoilage, which provides a quantitative basis for the color-change threshold of indicator films; second, a fundamental material innovation that replaces synthetic dyes with natural dyes, thereby addressing the biotoxicity issues of conventional indicators at the source; and third, the functional enhancement of natural dyes by leveraging advanced materials such as nanotechnology and metal–organic frameworks, effectively overcoming their inherent bottlenecks of poor stability and high susceptibility to migration.

Despite these notable advances, the technology still faces multiple challenges on its path toward commercial application, including insufficient resistance to environmental interferences, a lack of long-term reliability validation, the absence of standardized evaluation systems, and cost control issues in large-scale production. In particular, it should be noted that the excellent sensitivity and response rates attained under current laboratory conditions are often difficult to reproduce in large-scale extrusion coating or printing processes; process adaptability thus remains a critical bottleneck restricting industrialization.

Looking forward, future research in this field may prioritize breakthroughs in the following directions: first, establishing a standardized performance evaluation system for indicator films and promoting the initiation and formulation of relevant ISO or national standards, thereby providing a unified benchmark for cross-study comparisons and market regulation; second, exploring machine learning-assisted screening and formulation optimization of natural dyes, accelerating the shift from empirical trial-and-error to data-driven rational design; third, developing multifunctional integrated materials that combine biodegradability, indication functionality, and antibacterial preservation, so as to realize the systematic synergy of “packaging–indication–preservation.” Furthermore, the deep integration of indicator films with digital technologies such as the Internet of Things and smartphone-based spectroscopic analysis holds considerable promise for delivering more actionable food safety information to consumers.

Through cross-disciplinary technological integration and engineering optimization, smart indicator films are expected to evolve from a laboratory technology into a practical and reliable component of smart food packaging systems, ultimately providing technical support for building a safer, more efficient, and sustainable global food supply chain.

## Figures and Tables

**Figure 1:**
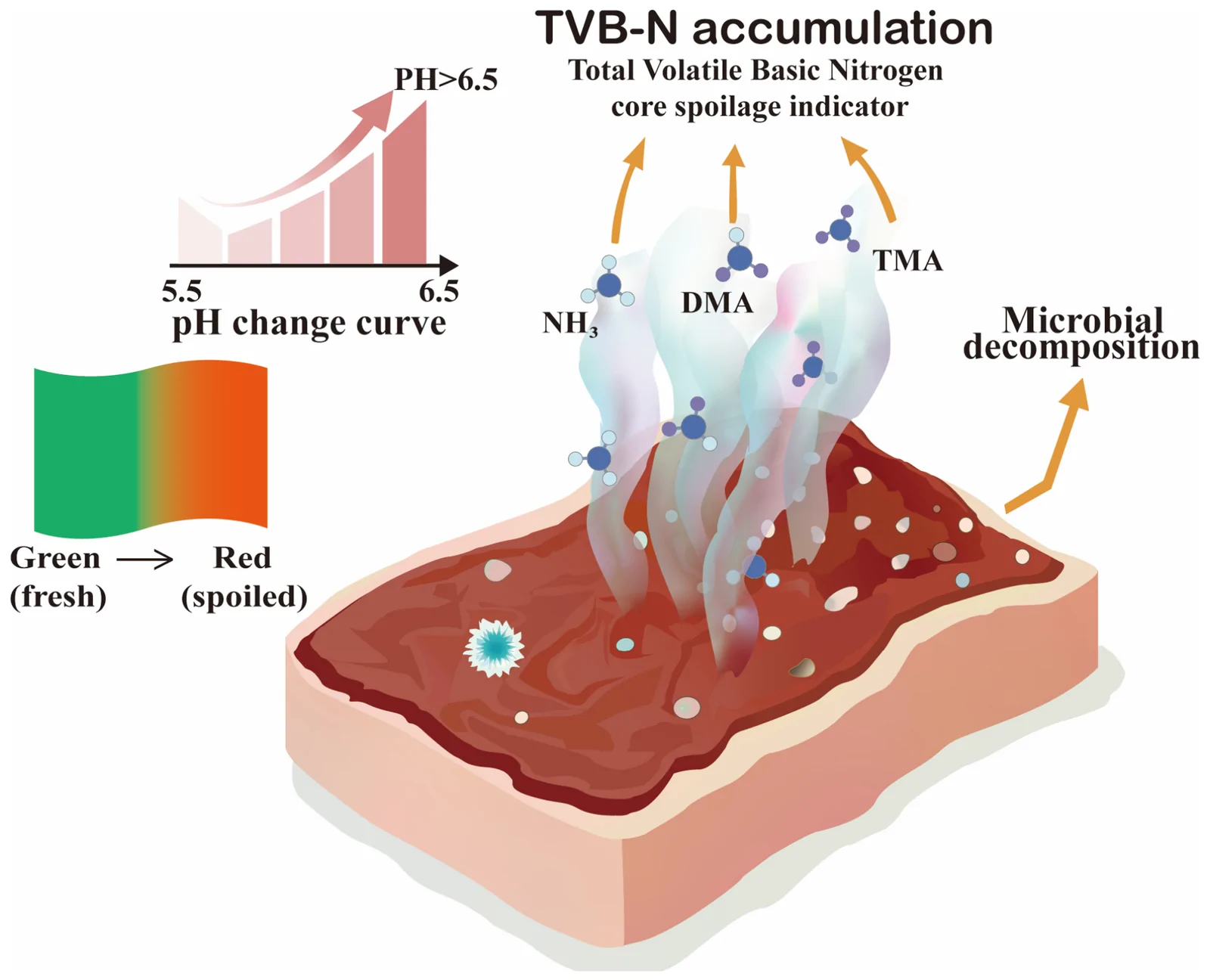
Conceptual illustration of the meat spoilage process and its relationship to pH-responsive indicator film operation. The diagram summarizes the cascade from microbial proliferation (origin) to TVB-N accumulation (chemical signature) and pH rise (physicochemical output) during meat storage. The rightmost panel indicates that the indicator film responds to headspace volatiles (e.g., NH3) through a localized pH change within its hydrated sensing layer, which in turn triggers a color transition. This schematic is intended as a conceptual framework to illustrate the general spoilage–indicator relationship; the specific pH value and color-change point are matrix-dependent and should be calibrated empirically against TVB-N, microbiological, and sensory thresholds for each particular food–packaging system, and are not presented as general constants. This illustration was prepared using Adobe IllustratorAdobe Illustrator CC 2019. The conceptual framework is based on the spoilage mechanisms described in references [16,31,34].

**Figure 2:**
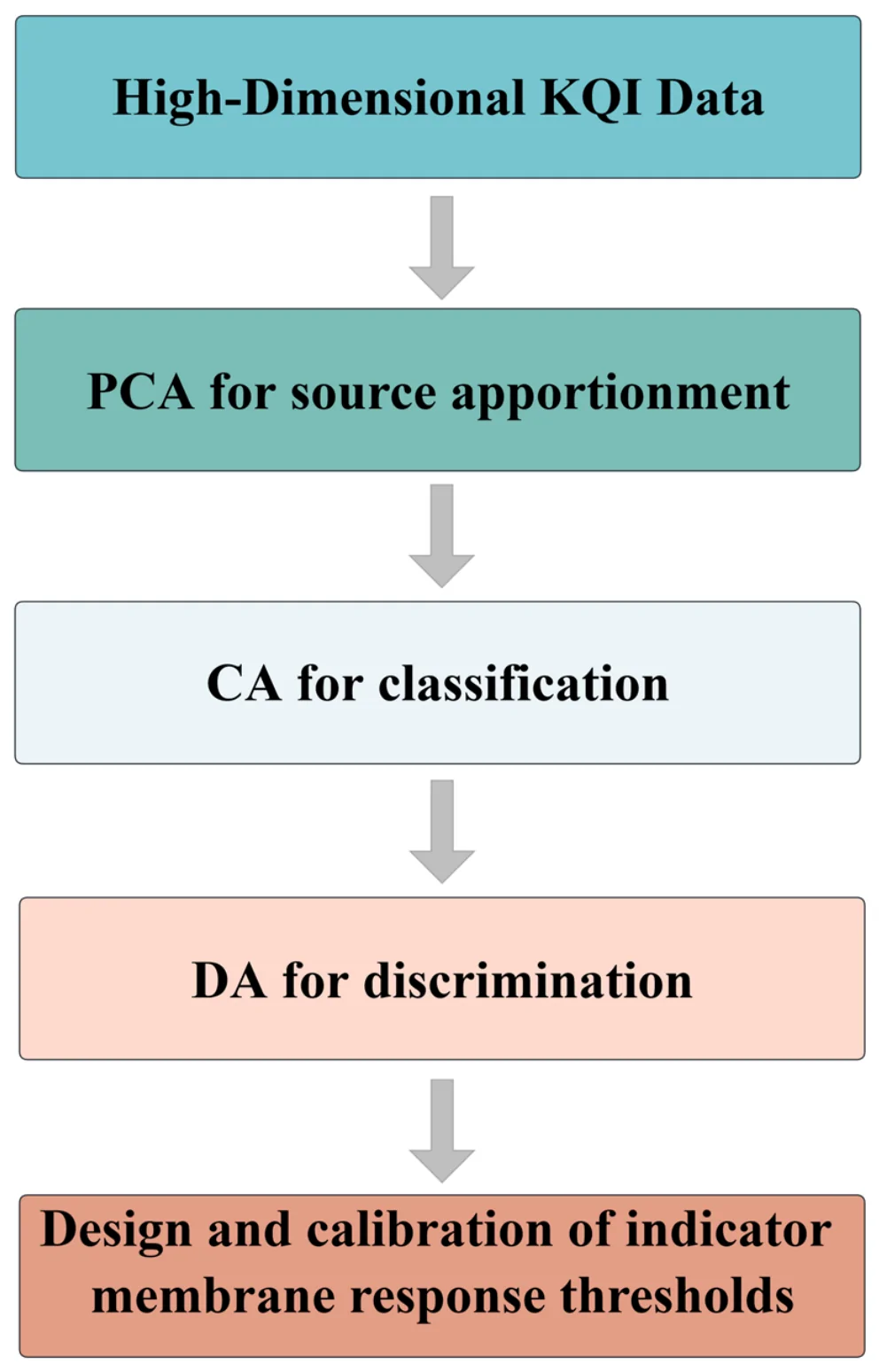
Schematic workflow of multivariate statistical analysis for food spoilage pattern recognition. This illustration was prepared using Adobe IllustratorAdobe Illustrator CC 2019. The analytical pipeline is adapted from the multivariate statistical frameworks described in [48,49,50].

**Figure 3:**
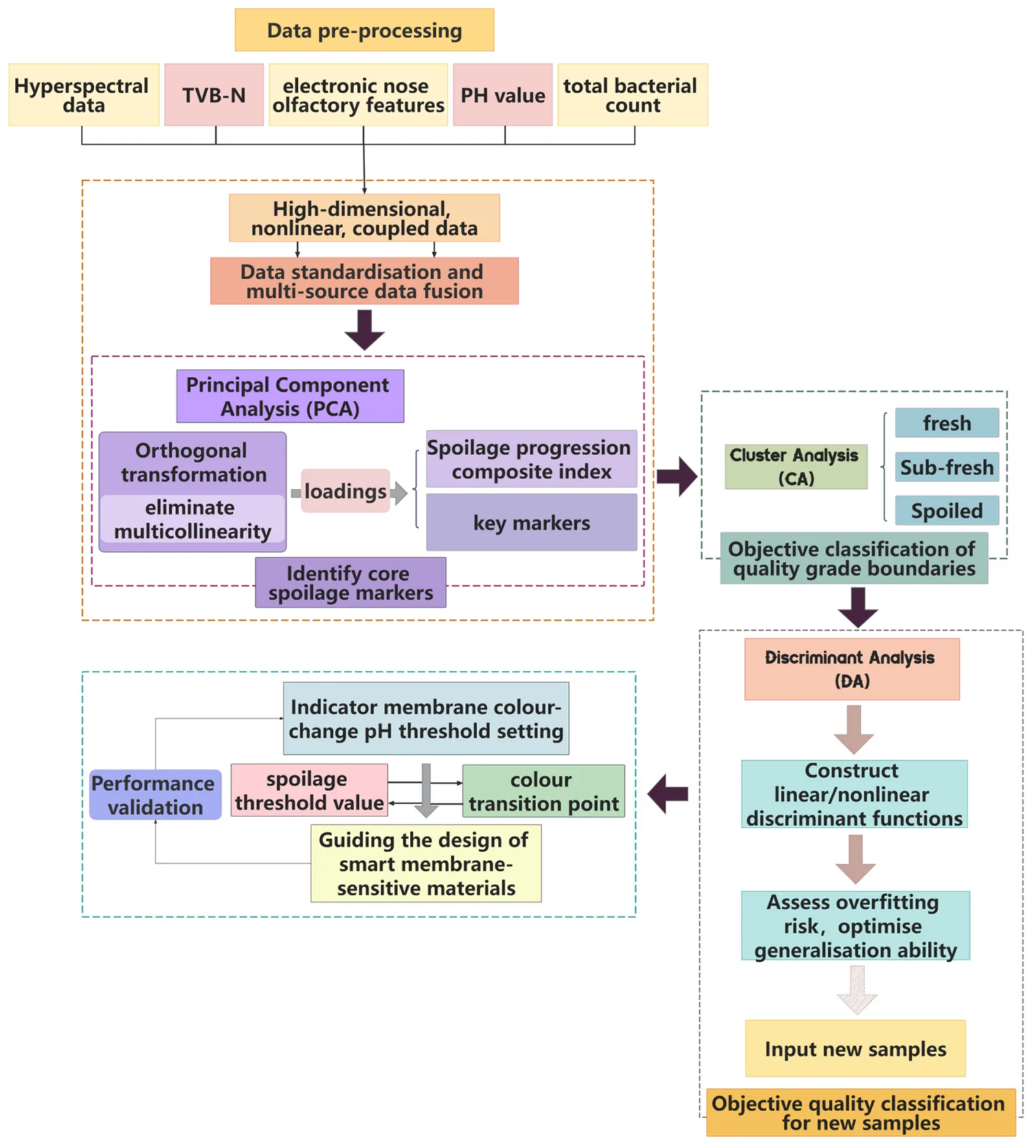
Flowchart of the PCA–CA–DA modeling procedure for indicator film threshold calibration. This illustration was prepared using Adobe IllustratorAdobe Illustrator CC 2019. The modeling workflow is based on the multivariate statistical approaches reviewed in references [47,50,53].

**Figure 4:**
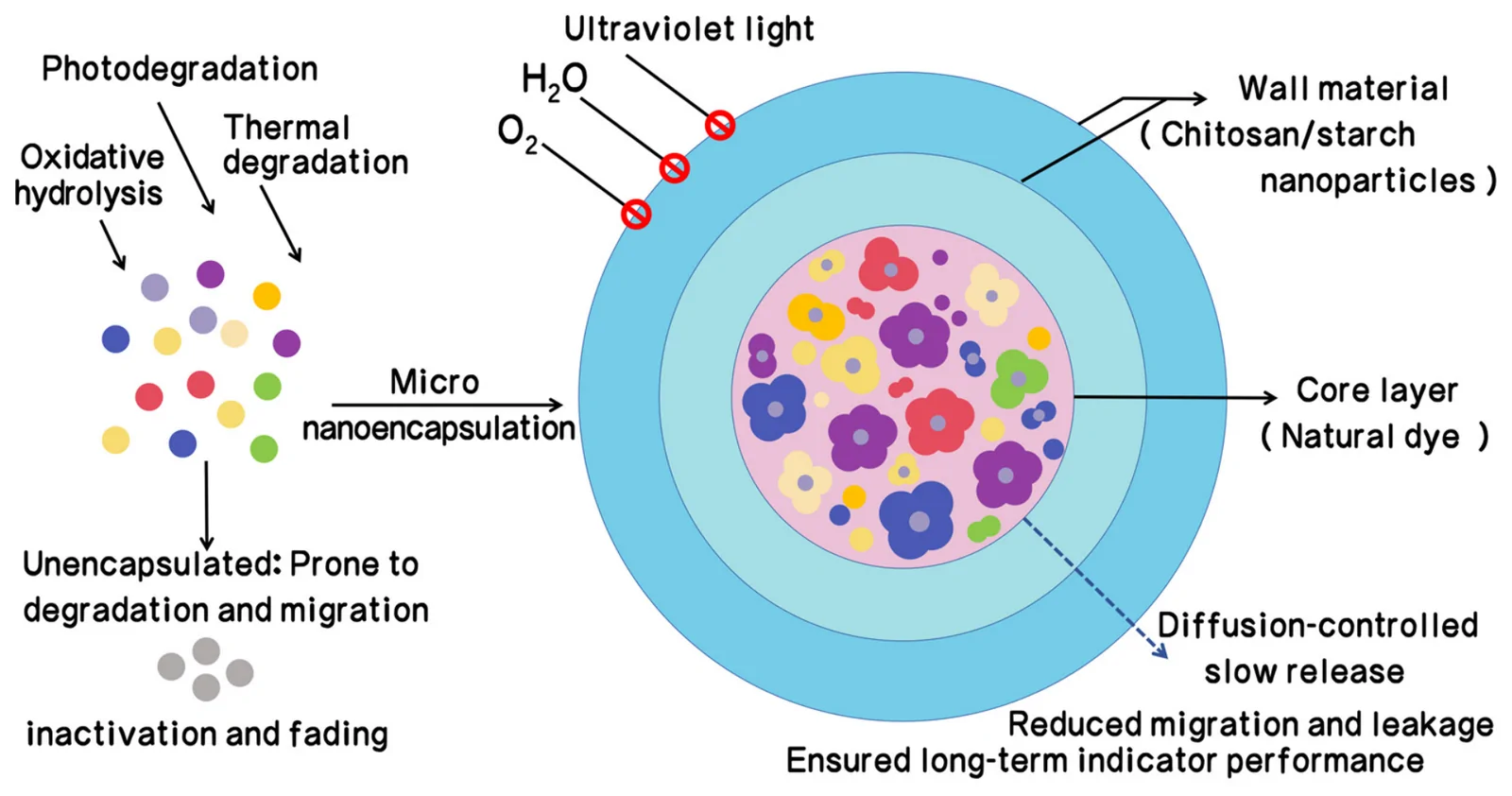
Schematic diagram of core--shell structured micro-/nano-encapsulation carriers. The core layer contains the natural dye, while the outer shell (wall material) provides physical protection and enables diffusion-controlled release. The distinct colors of the core and shell layers are used solely for visual clarity and do not carry specific compositional meaning. The symbol in the upper-right corner indicates that external factors—such as water, oxygen, and ultraviolet light—are blocked by the shell, thereby preventing degradation and migration of the encapsulated dye. This illustration was prepared using Adobe IllustratorAdobe Illustrator CC 2019. The encapsulation concept is based on the carrier systems described in references [66,67].

**Figure 5:**
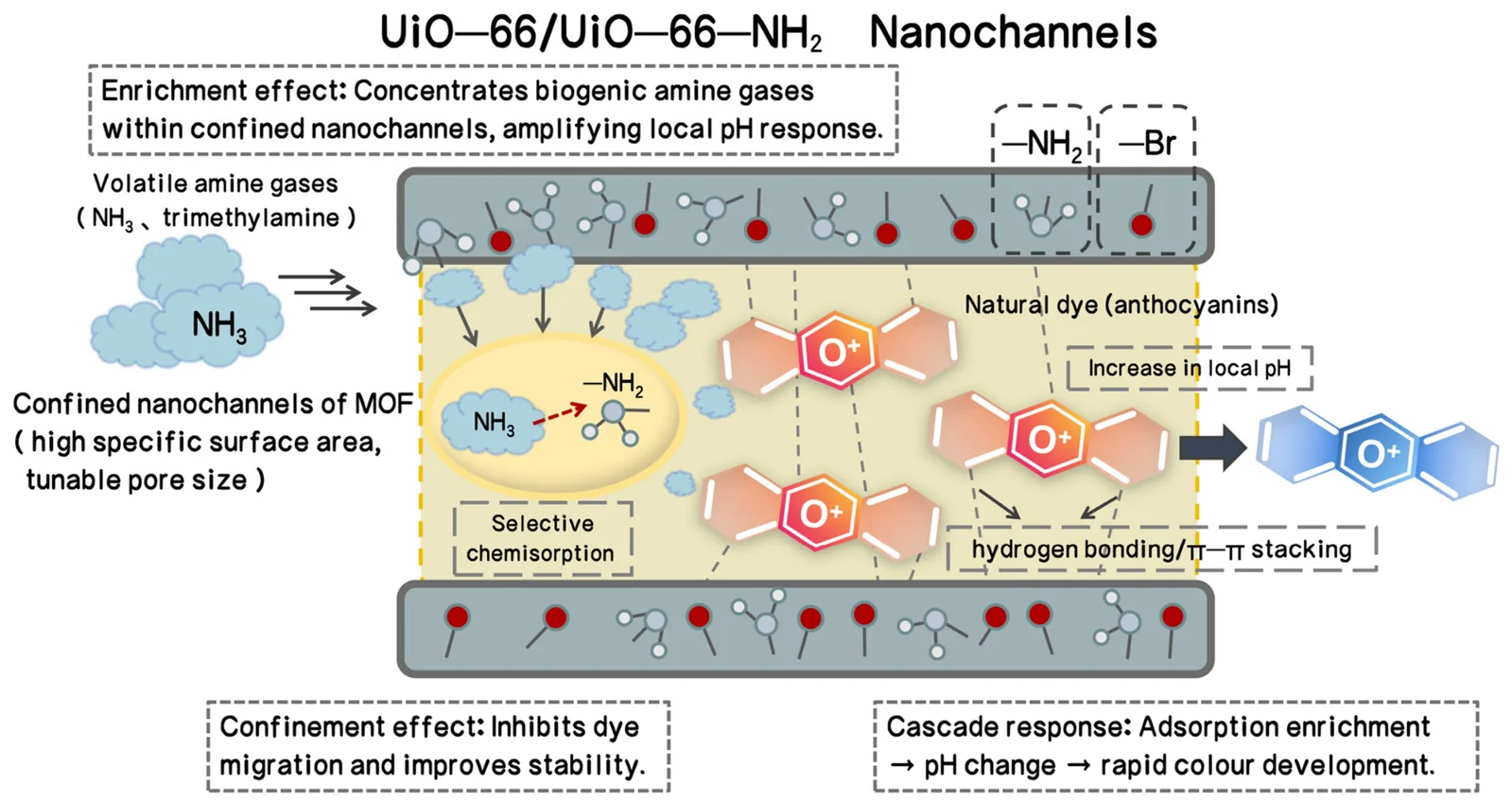
Schematic diagram of the structure of dye-immobilized MOFs. The MOF nanochannels (UiO-66 series) serve as hosts for natural dye molecules (anthocyanins) and provide spatial confinement. Volatile amine gases (NH_3_, trimethylamine) enter the nanochannels, are enriched via the enrichment effect, and trigger a localized pH increase. As the local pH rises from acidic to alkaline conditions, the natural dye molecules undergo a structural transformation, resulting in a color change from red to blue. The cascade response pathway (adsorption enrichment → pH change → color development) is indicated by the sequence of arrows. This illustration was prepared using Adobe IllustratorAdobe Illustrator CC 2019. The MOF immobilization concept is adapted from the dye@MOF composite systems reported in reference [24].

**Table 1:** Comparison of different types of intelligent packaging indicators (TTI, FI, and MGI) [6,8,17,18].

Indicator Type	Time-Temperature Indicator (TTI)	Freshness Indicator (FI)	Gas Indicator (MGI)
Monitoring Principle	Irreversible response based on chemical/enzymatic reaction kinetics to the cumulative time-temperature effect	Chemical recognition of specific spoilage metabolites (e.g., amines, sulfides, organic acids)	Chemical or enzymatic reactions with specific gases
Monitoring Target	Cumulative time-temperature effect of the storage/transport environment	Characteristic spoilage products (amines, sulfides, organic acids, etc.)	Headspace gas composition (O_2_, CO_2_ concentration) in the package
Core Advantage	Intuitively reflects the “thermal history” of the cold chain; high technological maturity	Directly indicates the actual quality status of food; enables non-destructive, real-time monitoring	Real-time monitoring of the package microenvironment; ensures the efficacy of modified atmosphere packaging
Fundamental Limitation	Does not directly reflect food quality; not applicable to non-temperature-sensitive spoilage	Limited generality across complex food matrices; sensitivity and stability require further optimisation	Does not directly reflect the food’s own spoilage degree; single-function
Typical Application Scenarios	Cold chain logistics monitoring; frozen foods; vaccine transport	Fresh meat; aquatic products; dairy products; ready-to-eat foods	Modified atmosphere packaging; fresh fruits and vegetables; baked goods

**Table 2:** Summary of material compositions and fabrication strategies for representative pH-responsive indicator films reviewed in this work.

Pigment Source	Film Matrix	Fabrication Strategy	Target Food	Mechanism Type	Color Change (pH Range)	Ref.
Black rice anthocyanins	Pectin/Chitosan	Solution casting	Pork	Acid–base equilibrium	Red → Purple → Yellow (pH 2–12)	[19]
Red cabbage anthocyanins	Bacterial cellulose	Solution casting	—	Acid–base equilibrium	Red → Green → Yellow (pH 2–10)	[11]
Butterfly pea flower extract	Sugarcane wax/Agar	Solution casting	Shrimp	Acid–base equilibrium	Pink → Purple → Green–Yellow (pH 2–12)	[20]
Black carrot anthocyanins	Chitosan/PVA/Clay	Solution casting (nanocomposite)	—	Acid–base + clay reinforcement	Red–Purple → Blue–Green (pH 2–12)	[21]
Anthocyanins + Shikonin (blend)	CMC/Agar	Solution casting (pigment blending)	Meat	Blended acid–base (dual pKa)	Red–Purple → Blue–Yellow (pH 2–12)	[22]
Anthocyanins + Curcumin (blend)	Carrageenan	Solution casting (pigment blending)	Chicken	Blended acid–base (dual pKa)	Enhanced contrast across pH 3–10	[23]
Anthocyanins loaded on UiO-66	PVA/Chitosan	Solution casting + MOF immobilization	Chicken	MOF confinement + acid–base	Red → Blue → Yellow (pH 2–12)	[24]
Curcumin + Salicylic acid	HPMC	Solution casting	Pork & Shrimp	Acid–base + antibacterial	Yellow → Orange–Red (pH 3–10)	[25]

Abbreviations: CMC, carboxymethyl cellulose; HPMC, hydroxypropyl methylcellulose; MOF, metal–organic framework; PVA, polyvinyl alcohol.

**Table 3:** Performance characteristics and key limitations of representative pH-responsive indicator films reviewed in this work.

Pigment Source (Matrix Reference)	Temp. (°C)	Response Characteristics	TVB-N Threshold (mg/100 g)	Stability & Migration	Key Limitations	Ref.
Black rice anthocyanins (Pectin/Chitosan)	4	pH 2–12, ~30–60 min, ΔE reported	N/A	Moderate (7 days at 4 °C); migration not reported	Narrow color contrast in neutral range	[19]
Red cabbage anthocyanins (Bacterial cellulose)	25	pH 2–10, ~15–30 min, L*a*b* reported	N/A	Moderate; migration not reported	Limited mechanical strength	[11]
Butterfly pea flower extract (Sugarcane wax/Agar)	25	pH 2–12, ~20–40 min, ΔE reported	~25	Moderate; migration not reported	High water solubility → dye leaching	[20]
Black carrot anthocyanins (Chitosan/PVA/Clay)	RT	pH 2–12, ~10–20 min, ΔE reported	N/A	Good (clay-enhanced); migration not reported	Complex clay dispersion	[21]
Anthocyanins + Shikonin blend (CMC/Agar)	4	pH 2–12, ~15–30 min, ΔE + L*a*b*	N/A	Moderate; migration not reported	Limited light stability; food-storage validation not detailed in the cited study	[22]
Anthocyanins loaded on UiO-66 (PVA/Chitosan)	4	pH 2–12, ~10–30 min, ΔE reported	~20	Good (MOF-enhanced); limited migration reported under specific simulant conditions (study-specific)	Metal ion/ligand migration not fully assessed	[24]
Curcumin + Salicylic acid (HPMC)	4	pH 3–10, ~30–60 min, ΔE reported	~22	Good thermal stability; migration not reported	Modest antibacterial effect; limited color variation	[25]

Abbreviations: RT, room temperature; ΔE, total color difference; N/A, not available or not reported in the cited study.

## Data Availability

No new data were created or analyzed in this study. Data sharing is not applicable to this article.

## Abbreviations

The following abbreviations are used in this manuscript:

BPANN	Backpropagation Artificial Neural Network
COF	Covalent Organic Framework
CS	Chitosan
FI	Freshness Indicator
HCA	Hierarchical Cluster Analysis
HPMC	Hydroxypropyl Methylcellulose
KQI	Key Quality Indicator
LDA	Linear Discriminant Analysis
MAP	Modified Atmosphere Packaging
MGI	Gas Indicator
MOF	Metal–Organic Framework
PCA	Principal Component Analysis
PLSDA	Partial Least Squares Discriminant Analysis
PVA	Polyvinyl Alcohol
TBARS	Thiobarbituric Acid Reactive Substances
TTI	Time-Temperature Indicator
TVB-N	Total Volatile Basic Nitrogen
TVC	Total Viable Count
ΔE	Color Difference
